# Simeprevir restores the anti-*Staphylococcus* activity of polymyxins

**DOI:** 10.1186/s13568-023-01634-8

**Published:** 2023-11-02

**Authors:** Yuan Wu, Pingyun Wu, Ruolan Wu, Huilong Li, Yao Duan, Chaoni Cai, Zixin Liu, Pengfei She, Di Zhang

**Affiliations:** Department of Laboratory Medicine, The Third Xiangya Hospital of Central South University, Changsha, Hunan 410013 China

**Keywords:** *Staphylococcus aureus*, Simeprevir, Polymyxins, Antimicrobial, Drug repurposing, Persister cells

## Abstract

Methicillin-resistant *Staphylococcus aureus* (MRSA) infection poses a severe threat to global public health due to its high mortality. Currently, polymyxins are mainly used for the treatment of Gram-negative bacterial-related infection, while exhibiting limited antibacterial activities against *Staphylococcus aureus* (*S. aureus*). However, the combination of antibiotics with antibiotic adjuvants is a feasible strategy for the hard-treated infection and toxicity reducing. We will investigate the antibacterial activity of simeprevir (SIM), which treated for genotype 1 and 4 chronic hepatitis C, combined with polymyxins against MRSA through high-throughput screening technology. In our study, the synergistic antibacterial effect of SIM and polymyxins against *S. aureus* in vitro was found by checkerboard assay and time-growth curve. The cytotoxicity of SIM combined with polymyxin B sulfate [PB(S)] or polymyxin E (PE) in vitro was evaluated using CCK-8, human RBC hemolysis and scratch assays. In addition, we investigated the eradication of biofilm formation of *S. aureus* by biofilm inhibition assay and the killing of persister cells. Moreover, we evaluated the therapeutic effect and in vivo toxicity of the combination against MRSA in murine subcutaneous abscess model. Furthermore, it was preliminarily found that SIM significantly enhanced the destruction of MRSA membrane by SYTOX Green and DISC3(5) probes. In summary, these results reveal that the therapy of SIM combined with polymyxins (especially PE) is promising for the treatment of MRSA infection.

**Supplementary Information:**

The online version contains supplementary material available at 10.1186/s13568-023-01634-8.

## Introduction

*Staphylococcus aureus* (*S. aureus*) is one of the main pathogens of hospital and community acquired infection (Diekema et al. 2019), which caused a variety of infectious diseases (including skin and soft tissue infection, endocarditis, severe sepsis and septicemia) (Lakhundi and Zhang 2018; Lowy 1998) with gradually increasing incidence from 1990 to 2019 (Vázquez-Sánchez et al. 2022). Since the 1960s, abuse of antibiotics and poor control measures of bacterial infection have led to the worldwide spread of MRSA which accounts for at least 25–50% among *S. aureus* (Lee et al. 2018; Vázquez-Sánchez et al. 2022). In most cases, MRSA resisted to most antibiotics (including tetracyclines, aminoglycosides, ß-lactams, etc.), of which the infection types range from asymptomatic nasal colonization to mild skin and soft tissue infection, further to fulminant invasive disease with high mortality, thus making clinical treatment difficult (David and Daum 2017; Lee et al. 2018). Moreover, the formation of biofilm enhances resistance to antimicrobial agents and immune defenses, playing an important role in persistent chronic infection(Schilcher and Horswill 2020). Persister cells, randomly formed during biofilm growth, have reduced metabolic activity and exhibited high resistant to all available antibiotics, making the infection difficult to be eradicated and prone to recurrent infection (Fisher et al. 2017; Rowe et al. 2021). Therefore, there is an urgent need for new antimicrobial agents to combat MRSA and its biofilm and persister cells-related infection. However, new drugs discovery consumes a lot of labor and time. Drug repurposing or combinational therapy is a more feasible strategy than the traditional drug development.

Polymyxins, of which polymyxin B (PB) and polymyxin E (PE) are commonly used in clinical settings (Dai et al. 2020), are currently the last line of drugs in the treatment of multidrug-resistant (MDR) Gram-negative bacterial infection (Bian et al. 2022), but their high nephrotoxicity and neurotoxicity remain concerns (Nang et al. 2021). The bactericidal activities of polymyxins against Gram-negative bacteria are mainly dependent on the electrostatic interactions between positively charged polymyxins and negatively charged lipopolysaccharide (LPS) (Li and Velkov 2019; Moffatt et al. 2019). So, structural changes in LPS are currently common causes of resistance to polymyxins in Gram-negative bacteria (Carretero-Ledesma et al. 2018; Moffatt et al. 2019). However, it is difficult to treat MRSA with polymyxins alone due to the lack of LPS in Gram-positive bacteria (Yin et al. 2020).

The small macrocyclic drug simeprevir (SIM) was first approved in 2013. SIM is recommended for the treatment of genotype 1 and 4 chronic hepatitis C in combination with peginterferon and ribavirin, which was reported with low incidence of clinical adverse events (including rash and anemia) (Vaidya and Perry 2013; You and Pockros 2013). SIM acts by inhibiting the viral NS3/4A serine protease and blocking replication of hepatitis C virus in host cells (Vaidya and Perry 2013). In 2022, SIM was selected from a library of 1573 drugs approved by U.S. Food and Drug Administration to explore its antibacterial activity against *S. aureus* through high-throughput screening assays (Li et al. 2022). However, to the best of our knowledge, there is no report about the combinational antimicrobial effect between SIM and polymyxins against *S. aureus*.

In our study, we firstly found the synergistic antibacterial effect of SIM combined with polymyxins (especially PE) against MRSA in vitro and in vivo. Then, the anti-biofilm and anti-persister cells activities of the combination were further explored. The mechanisms of SIM restored the anti-*S. aureus* effects of polymyxins mainly involved in cell membrane disruption. In addition, SIM combined with polymyxins showed extremely low toxicity in vitro and in vivo. The combination of antibiotics with antibiotic adjuvants is an effective treatment option for the hard-treated infection and toxicity reducing (Douafer et al. 2019; Tyers and Wright 2019).

## Materials and methods

### Reagents, strains and culture conditions

Type strains used in this study were shown in Table 1. Clinical strains of *S. aureus* and *Enterococcus faecium* were isolated from the Third Xiangya Hospital of Central South University, and identified by VITEK 2 Compact (bioMerieux, France) as well as Matrix-Assisted Laser Desorption Ionization (BD, Germany). *S. aureus* and *S. epidermidis* were grown in Tryptic Soy Broth (TSB) (Solarbio, Beijing, China). Simeprevir (SIM), polymyxin B sulfate [PB(S)], colistin (polymyxin E, PE), ciprofloxacin (CIP) and other antimicrobials were purchased from the MedChem Express (New Jersey, USA) and dissolved in deionized water or dimethyl sulfoxide (DMSO).

**Table 1:** Bacterial strains used in this study

Bacterial species	Strains	Source
*S. aureus*	ATCC 25,923	Juncai Luo^a^
	ATCC 29,213	Juncai Luo^a^
	ATCC 43,300	Min Li^b^
	USA300	Min Li^b^
	MW2	Min Li^b^
*Enterococcus faecalis*	ATCC 29,212	Juncai Luo^a^
*Pseudomonas aeruginosa*	PAO1	Qiao Minqiang^c^

a. Tiandiren Biotech, Changsha, China. b. Renji Hospital, School of Medicine, Shanghai Jiao Tong University. c. College of Life Sciences of Nankai University, Tianjin, China.

### Antimicrobial susceptibility test

The minimum inhibitory concentrations (MICs) of the antibiotics used in this study were determined by the standard microdilution method prescribed by the Clinical & Laboratory Standards Institute(CLSI 2023). Briefly, overnight cultured bacterial suspension was diluted to 1.5 × 10^6^ CFU/mL. The bacterial suspension was mixed with the serially diluted antimicrobials in equal volume into a 96-well plate, further incubated at 37℃ for 16 ~ 18 h, and the concentration at which no visible bacterial growth was defined as MIC. After the bacterial suspension cultured on 5% sheep blood agar plate (Autobio, Zhengzhou, China) for 24 h, the concentration that kills 99.9% of the colonies was defined as the minimum bactericidal concentration (MBC).

### Checkerboard assay

Checkerboard assay was used to assess the antimicrobial synergies between two drugs. Briefly, equal volumes of 2-fold diluted SIM and polymyxins by Mueller–Hinton (MH) II broth (Solarbio, Shanghai, China) were added to a 96-well plate in vertical and horizontal order, respectively, in the presence of 1 × 10^6^ CFU/mL *S. aureus*. After incubation at 37℃ for 16–18 h, the optical density at 630 nm (OD_630_) was measured, and the antibacterial interaction between the drugs was calculated by fractional inhibition concentration index (FICI) as follows: ${\rm{FICI}}\,{\rm{ = }}\,\frac{{{\rm{MIC}}A\,{\rm{combination}}}}{{{\rm{MIC}}A\,{\rm{alone}}}}\,{\rm{ + }}\,\frac{{{\rm{MIC}}B\,{\rm{combination}}}}{{{\rm{MIC}}B\,{\rm{alone}}}}{\rm{.}}$ FICI ≤ 0.5 indicates synergy, 0.5 < FICI ≤ 4 indicates no interaction, and FICI > 4 indicates antagonism (She et al. 2022).

### Kirby-Bauer test

The single colony of *S. aureus* was adjusted to a McFarland (McF) turbidity of 0.5 with sterile saline and spread on MH agar plate. Then the sterile discs containing the indicated concentrations of antimicrobials were placed on the MH agar plate. The diameters of the inhibition zones were measured after incubation at 37℃ for 18 h (Ul Haq et al. 2022).

### Time-growth inhibition assay

A single colony of *S. aureus* was inoculated into an appropriate amount of TSB broth to log phase. The bacterial solution was adjusted to 1 × 10^6^ CFU/mL. SIM and polymyxins alone or in combination was added to the corresponding bacterial suspension to the indicated concentration. DMSO was used as a control group. Then, the bacterial suspension was incubated at 37 °C 180 rpm, an aliquot of the bacterial suspension in each group was removed into a 96-well plate for the detection of OD_630_ at the time point of 0, 0.5, 1, 2, 4, 6, 8, and 12 h, respectively (She et al. 2021).

### Dead/live bacterial cells quantification by SYTO9/PI staining

Log-phase-grown ATCC 43,300 was diluted with TSB to 1 × 10^6^ CFU/mL. SIM (1 µg/mL) alone or in combination with PB(S) (16 µg/mL) or PE (4 µg/mL) was added to the bacterial suspension, respectively. After incubated at 37 °C 180 rpm for 4 h, the bacterial suspension was added with 10 µM of SYTO9 and PI mixed solution. After stained for 10 min in dark, the bacterial precipitation was collected by centrifugation and re-suspended in sterile saline and further observed by a fluorescence microscope (Zeiss Vert A1). The excitation and emission wavelength of SYTO9 was 488 nm and 550 nm, respectively, and those of PI was 540 nm and 620 nm, respectively (She et al. 2022).

### Resistance inducing assay

The MICs of polymyxins alone or in combination with SIM against *S. aureus* ATCC 43,300 and USA300 were determined by the antimicrobial susceptibility test as described above. Then, the bacterial suspension at 1/2×MIC was 1:1000 diluted with MH broth and further used to perform the antimicrobial susceptibility test for the next day. The assay was consecutively performed for 7 days, and the MIC value was recorded daily (She et al. 2021).

### Persister cells killing assay

MRSA were induced to the stationary-phased persister cells after cultured at 37 °C and 200 rpm for 24 h, and adjusted to a 1 × 10^8^ CFU/mL(Li et al. 2022). Then indicated concentrations of SIM and polymyxins alone or in combination were added into the bacterial suspension. After incubation at 37 °C, 200 rpm for 4 h, the live persister cells were calculated by serial dilution and CFU counting.

### Human RBC hemolysis

Human RBC was purchased from the Hemo Pharmaceutical and Biological Co (Shanghai, China). After centrifugation at 1000 g for 5 min, the RBC pellets were resuspended in 5% (vol/vol) sterile saline with equal volumes of indicated concentrations of polymyxins in combination with 4 µg/mL of SIM and further incubated at 37 °C for 1 h. After centrifugation at 1000 g for 5 min, the supernatants were transferred to a 96-well plate, and the absorbance at 570 nm (A_570_) was measured. 0.12% DMSO and 1% TritonX-100 were used as negative and positive control, respectively (Tan et al. 2020). The hemolysis rate was calculated as follows: ${\rm{Hemolysis}}\,{\rm{(\% )}}\,{\rm{ = }}\,{\rm{(}}\frac{{A{\rm{sample - ADMSO}}}}{{A{\rm{TritonX - 100 - ADMSO}}}}{\rm{)}}\,{\rm{ \times }}\,{\rm{100\% }}$.

### Cytotoxicity detection by CCK-8 kit

The cytotoxicity of SIM combined with polymyxins against 293T (Human renal epithelial cell line), HSF (Human skin fibroblast cell line) and HaCaT cell line (human immortalize epidermal cell line) was detected by CCK-8 assay. For example, 293T cells were cultured in DMEM medium (Kaiji Biotechnology Development Co, Nanjing, China) (containing 10% FBS + 1% double antibody) at 37℃, 5% CO_2_ in a saturated humidity incubator. 293T cells in logarithmic growth phase were seeded into a 96-well plate with about 2 × 10^3^ cells per well, and treated with indicated concentrations of antimicrobial-containing medium for 24 h. Fresh medium containing CCK-8 solution (including 90 µL complete medium and 10 µL CCK-8 solution) was added to each well. After incubation for 1.5 h at 37℃, the absorbance at 450 nm was measured by microplate reader (Luo et al. 2021).

### Scratch assay

We performed scratch assay as previously described (Lin et al. 2018; Luo et al. 2021). We plated HaCaT cells in 12-well plates, allowed them to adhere and grow to 90% confluence. Then, cells were scratched using a 200-µl pipette tip to create a scratch wound area and washed gently with phosphate buffer saline (PBS) twice to remove detached cells. And culture medium in the presence of SIM and polymyxins alone or in combination were applied for 0, 12, and 24 h, respectively, and observed by an inverted microscope (Zeiss Vert A1). Cell migration ability was evaluated by the percentage of migration rate (distance migrated/original wound distance ×100%).

### Apoptosis detection by Annexin V-FITC/PI staining

The 293T cells were cultured as described above. After 4 h of cell attachment, the cells were incubated with drug-containing complete medium at 37℃ 5% CO_2_ for 24 h. Next, each well was washed 1–2 times with PBS and added with 500 µL of EDTA free trypsin for digestion. The cells were gently pipetted to single cell suspension, and the supernatant was discarded after centrifugation at 1000 g for 5 min. The cells were resuspended with 195 µL binding buffer, 5 µL Annexin V-FITC, and 10 µL PI and incubated for 10–20 min at room temperature in the dark. The controls were set as unstained group, PI staining group and Annexin V-FITC staining group, respectively. The fluorescence intensity was detected by flow cytometry with excitation/emission wavelengths of 488 nm/525 nm and 535 nm/615 nm for FITC and PI, respectively(BD, USA) (Liu et al. 2018).

### Calcein-AM/PI staining

The 293T cells were cultured and treated as described above. Each well was added with 500 µL of complete medium containing Calcein AM/PI (2/10 µM) solution. After incubation at 37℃ 5% CO_2_ for 30 min in the dark, the medium was replaced with fresh 37ºC preheated medium, and further incubated in the dark to ensure that the cell lactonase fully hydrolyzed Calcein AM to generate Calcein with green fluorescence. After washed twice with PBS, the cells were observed by the fluorescence microscope (Spark 10 M) with the maximum excitation wavelength of 494 nm/535 nm for Calcein/PI (Liu et al. 2018).

### Neutrophils intracellular killing

Neutrophils intracellular killing assay was used to determine the intracellular killing effect of SIM combined with polymyxins against *S. aureus*. Heparin-anticoagulated blood of healthy volunteers was mixed with an equal volume of Gibico 1640 medium, and pure neutrophils were separated from the blood by lymphocyte separation solution and erythrocyte lysis solution. The precipitation was resuspended with Gibico 1640 medium containing 2% fetal bovine serum and prepared to 3 × 10^6^ cells/mL. The stationary-phase USA300 bacterial suspension was adjusted to a McF of 0.5. The neutrophil suspension and bacteria solution of equal volume were added into each well of 96-well plate, and cultured in a saturated humidity incubator at 37℃ with 5% CO_2_ for 30 min. Gentamicin (final concentration: 50 µg/mL) was added to each well for 1 h incubation to remove the extracellular bacteria. The precipitation was resuspended in 100 µL of the corresponding drug-containing medium for each group. 0.1% TritonX-100 at 37℃ for 10 min was used to lysis the cells. Finally, the suspensions of different groups were diluted in gradient, and counted on 5% sheep blood agar plates (Fan et al. 2021; Kang et al. 2019).

### Biofilm inhibition assay

Similar as the above described checkerboard assay, equal volumes of 2-fold diluted SIM and polymyxins were added to a 96-well plate in vertical and horizontal order, respectively, in the presence of 1 × 10^6^ CFU/mL *S. aureus*. After 24 h incubation at 37 °C, the unattached cells were removed by PBS washing and further stained with 2% (wt/vol) crystal violet solution for 5 min. The unattached dye was removed and washed twice with PBS, and the A_570_ was measured and the plate was photographed(Zhang et al. 2021).

### Cell membrane disruption detection by SYTOX Green/DiSC3(5) staining

Log-phase-grown of *S. aureus* ATCC 43,300 was adjusted to an OD_630_ of 0.05 by 5 mM HEPES. For SYTOX Green staining, 2 µM of SYTOX Green was added to the bacterial suspension. Then indicated concentrations of SIM and polymyxins alone or in combination were added into the bacterial suspension. The fluorescence intensity was detected every 2 min for a total of 20 min at the excitation and emission wavelengths of 485 nm and 525 nm, respectively. For DiSC3(5) staining, the bacterial suspension with OD_630_ of 0.05 was mixed with 2 µM KCl, 5 mM glucose and 2 µM DiSC3(5). After incubation at room temperature in the dark for 1 h, different concentrations of polymyxins and SIM alone or in combination were applied in the bacterial suspension. The fluorescence intensity was detected every 30 s for 5 min at the excitation/emission wavelength of 622 nm/670 nm, respectively (Liu et al. 2021).

### Intracellular ROS quantification by 2′,7′-Dichlorofluorescin diacetate (DCFH-DA)

Overnight cultured *S. aureus* was centrifuged to remove the supernatant, and the cell precipitation was resuspended in 1×PBS to an OD_630_ of 0.5. The bacterial suspension was incubated with DCFH-DA at a final concentration of 10 µmol/L for 30 min, and then washed twice with PBS. The probe-labeled bacterial suspension and different groups of drugs in equal volume were added to 96-well plate and incubated for 30 min. The fluorescence intensity was measured at the excitation/emission wavelengths of 488 nm/525 nm, respectively (Liu et al. 2021).

### In vivo

All animals were purchased from Hunan SJA Laboratory Animal Co. Ltd. (Changsha, China). Six-week-old female ICR mice weighing approximately 25 ± 3 g were used in this study. The murine subcutaneous abscess model was established as previously reported by Pletzer et al. (Pletzer et al. 2018) with minor modifications. Briefly, the hair on the back was shaved using an animal electric razor. Log-phase-grown MRSA strain ATCC 43,300 was washed twice with saline and adjusted to 0.5 McF. One hundred microliter of the bacterial suspension was injected subcutaneously on the back. After 30 min of inoculation, a single dose of PB(S)/PE (30 mg/kg) and SIM (20 mg/kg) were subcutaneously injected alone or in combination. The mice injected with 1% DMSO were used as the vehicle group. Twenty-four hours post-infection, the abscess was excised and homogenized with sterile saline. The viable bacterial cells were counted by fold dilution as described above. Meanwhile, the skin specimens were fixed in 4% paraformaldehyde (Servicebio, Wuhan, China) and then subjected to hematoxylin and eosin (H&E) staining. To assess the in vivo toxicity of the SIM and polymyxins in combination, the blood samples were collected from the orbital vein, and the level of organic function biomarkers [including creatine kinase (CK), alanine aminotransferase (ALT) and creatinine (CREA)] were determined by Hitachi 7600 series automated biochemistry analyzer. Meanwhile, the heart, liver, lung, kidney and spleen were taken for H&E staining on the second day after infection.

### Statistical analysis

All experiments were performed independently in triplicate. All data were analyzed by GraphPad Prism 8.0 software and expressed as mean ± standard deviation (SD). Significant differences between two groups of data were compared using Student’s t-test, while data comparisons of more than two groups were performed using one-way ANOVA and Dunn’s multiple comparison test. *P*-value < 0.05 was considered statistically significant.

## Results

### Synergistic antimicrobial activity between SIM and PB(S)/PE against *S. aureus*

**Fig. 1:**
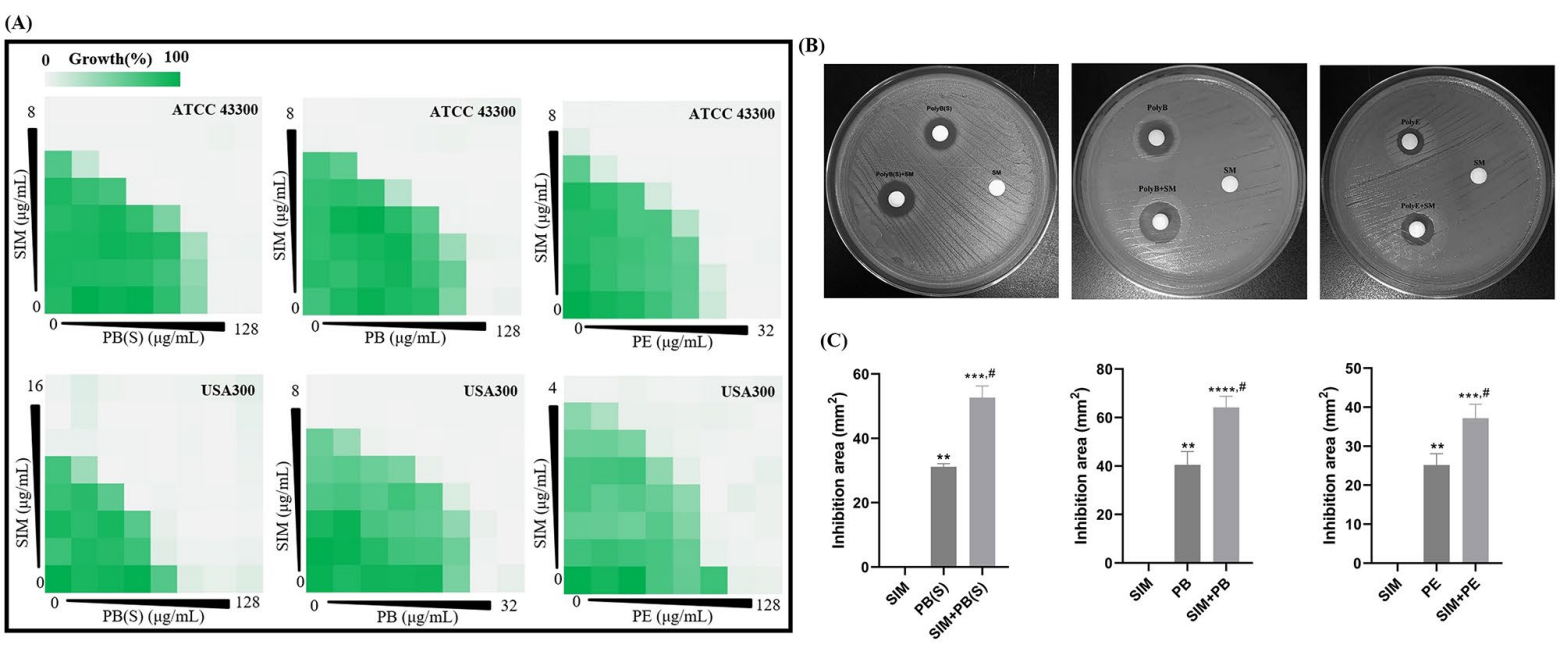
Antimicrobial synergy between SIM and polymyxins against *S. aureus.* (**A**) Antimicrobial effects of SIM combined with polymyxins against *S. aureus* ATCC 43,300 and USA300 by checkerboard assay. (**B**) Growth inhibition of antimicrobials alone or in combination against ATCC 43,300 determined by K-B test. PB: 250 µg; PB(S): 200 µg; PE: 200 µg. (**C**) Statistical analysis of the diameters about the inhibition zones by the K-B test. **: *P* < 0.01 compared with SIM; ***: *P* < 0.001 compared with SIM; ****: *P* < 0.0001 compared with SIM; *#*: *P* < 0.05 compared with PB(S)/PE.

**Fig. 2:**
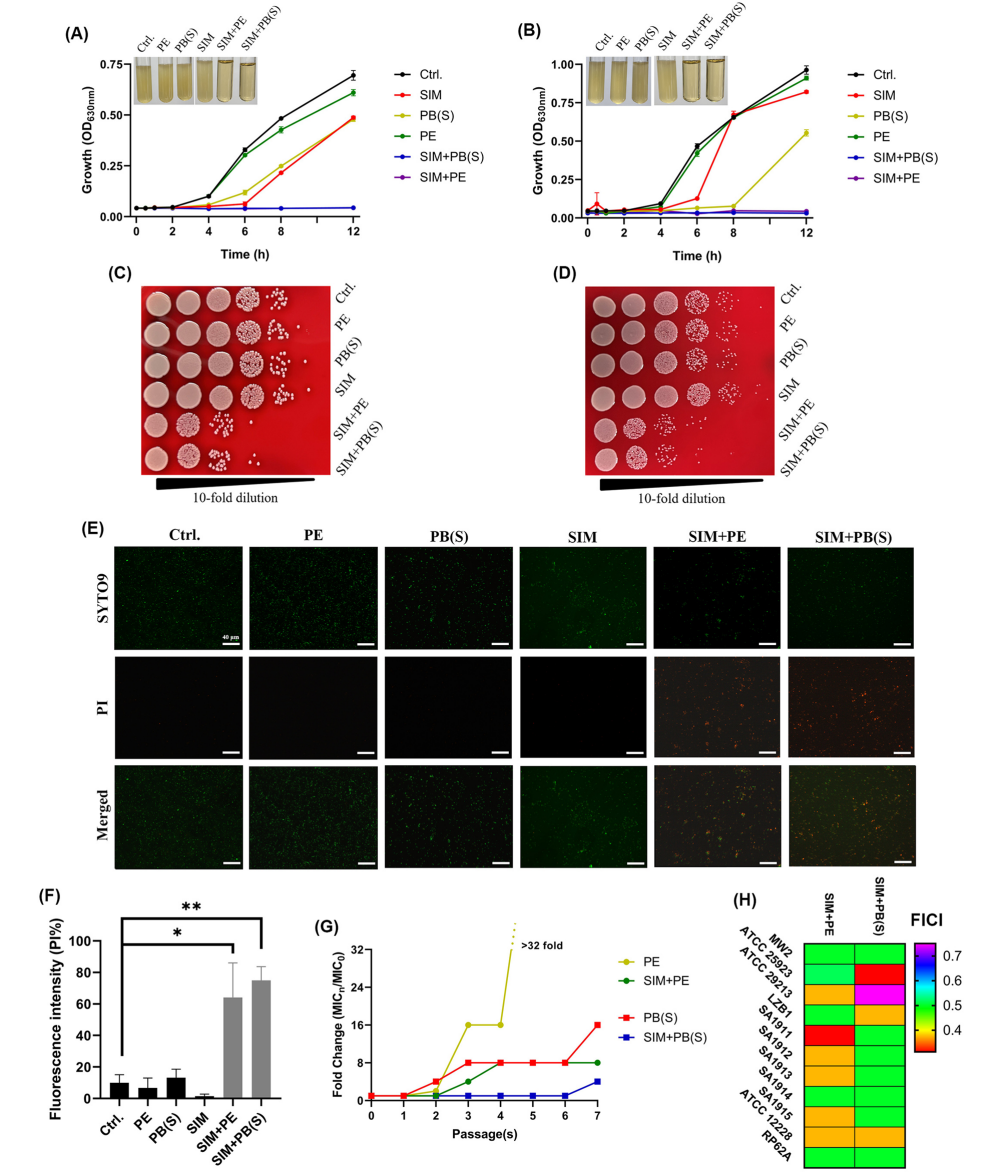
Synergistical bactericidal activity between SIM and polymyxins against *S. aureus*. Time-inhibition curves of SIM (1 µg/mL) and polymyxins (8 µg/mL) alone or in combination against USA300 (**A**) and ATCC 43,300 (**B**), respectively. Viable cells counting at the time point of 12 h at the highest gradient concentrations that can be well-counted for USA300 (**C**) and ATCC 43,300 (**D**), respectively. (**E**) Representative images of anti-planktonic bacterial effect of 1 µg/mL SIM alone or combined with 16 µg/mL PB(S) or 4 µg/mL PE against ATCC 43,300 by SYTO9 (green) and PI (red) staining. (**F**) Quantification of SYTO9/PI fluorescence intensity. (**G**) Sequential passaging resistance development of *S. aureus* USA300 treated with PB(S)/PE in the presence of sub-MIC (0.5 µg/mL) of SIM; (**H**) Drug combination between SIM and polymyxins against type strains and clinical isolates. *: *P* < 0.05; **: *P* < 0.01

We assessed the combinational antimicrobial effects between SIM and polymyxins or their derivatives [including PE, polymyxin B(PB), PB(S), PMBN, SPR741 and SPR206] against MRSA by checkerboard assay. The results showed that SIM could be synergistic with PB, PB(S) and PE against MRSA with FICI ≤ 0.5, although *S. aureus* exhibited intrinsic resistance to polymyxins with MIC ≥ 16 µg/mL due to its obstruction by outer membrane (Yin et al. 2020) (Table 2; Fig. 1A). However, other polymyxin derivatives, such as PMBN, SPR741 and SPR206, showed no interaction against MRSA when combined with SIM (Figure S1). The combinational antimicrobial activity was not observed in Gram-negative strains including *A. baumannii*, *E. coli*, *K. pneumoniae*, or *P. aeruginosa* (Figure S2). Further, K-B test was used to confirm the synergy between SIM and polymyxins. As shown in Fig. 1B and C, SIM in combination with PB(S) or PE formed a more significant growth inhibition zone against *S. aureus* than used alone. In addition, we found that the synergistic antimicrobial effects between SIM and PB(S)/PE against *Staphylococcus* type strains or clinical isolates (including MRSA, MSSA, and *S. epidermidis*) were strain independent with FICI ≤ 0.5 (Fig. 2H; Figure S3).

**Table 2:** SIM combined with polymyxins against MRSA

Strains	Antimicrobials	MIC _Alone_(µg/mL)	MIC _In combination_(µg/mL)	FICI	Outcome
ATCC 43,300	PB(S)	64	8	0.375	Synergy
	SIM	4	1		
	PB	64	16	0.5	Synergy
	SIM	4	1		
	PE	16	4	0.5	Synergy
	SIM	4	1		
USA300	PB(S)	32	8	0.5	Synergy
	SIM	4	1		
	PB	16	4	0.5	Synergy
	SIM	4	1		
	PE	64	8	0.375	Synergy
	SIM	4	1		

PB(S): polymyxin B sulfate; SIM: simeprevir; PB: polymyxin B; PE: polymyxin E

### SIM enhanced the bactericidal activity and reduced resistance occurrence of polymyxins

To further confirm the synergistic antibacterial effects between SIM and PB(S)/PE, we carried out time-growth inhibition and time-killing assay. According to the results of time-growth curve, sub-MICs of SIM (1 µg/mL), PB(S) (8 µg/mL) or PE (8 µg/mL) alone did not inhibit the growth of MRSA USA300 within 12 h, while synergistical inhibition effects were observed by SIM in combination with PB(S)/PE (Fig. 2A). Similarly, the synergistic bacterial growth inhibition effect was also found in *S. aureus* ATCC 43,300 (Fig. 2B). The representative images of viable CFU counts on sheep blood agar plates indicated the combination groups of PB(S)/PE with SIM had synergistic bactericidal activities against *S. aureus* (Fig. 2C and D). Further, the SYTO9/PI staining images showed that as compared to the control or monotherapy groups, the viable bacteria in the combined groups were markedly decreased (Fig. 2E). After quantification, the percentage of dead cells in the combination group was more than 60%, which was at least 5 times higher than that in the control group (accounting for ~ 10%) (*P* < 0.05) (Fig. 2F). Next, we studied the bacterial resistance induced by PB(S)/PE could be reduced in the presence of sub-MIC of SIM by calculating the fold change of MIC in a 7-day consecutive resistance induce. As shown in Fig. 2G, PE alone had at least a 32-fold increase in the values of MIC, whereas the MIC of PE only had an 8-fold increase in the presence of sub-MIC (0.5 µg/mL) of SIM against to *S. aureus* USA300. Similarly, the MIC of PB(S) exhibited 4-fold more decrease in the presence of SIM than PB(S) alone.

### Acceptable cytotoxicity of SIM combined with polymyxins

**Fig. 3:**
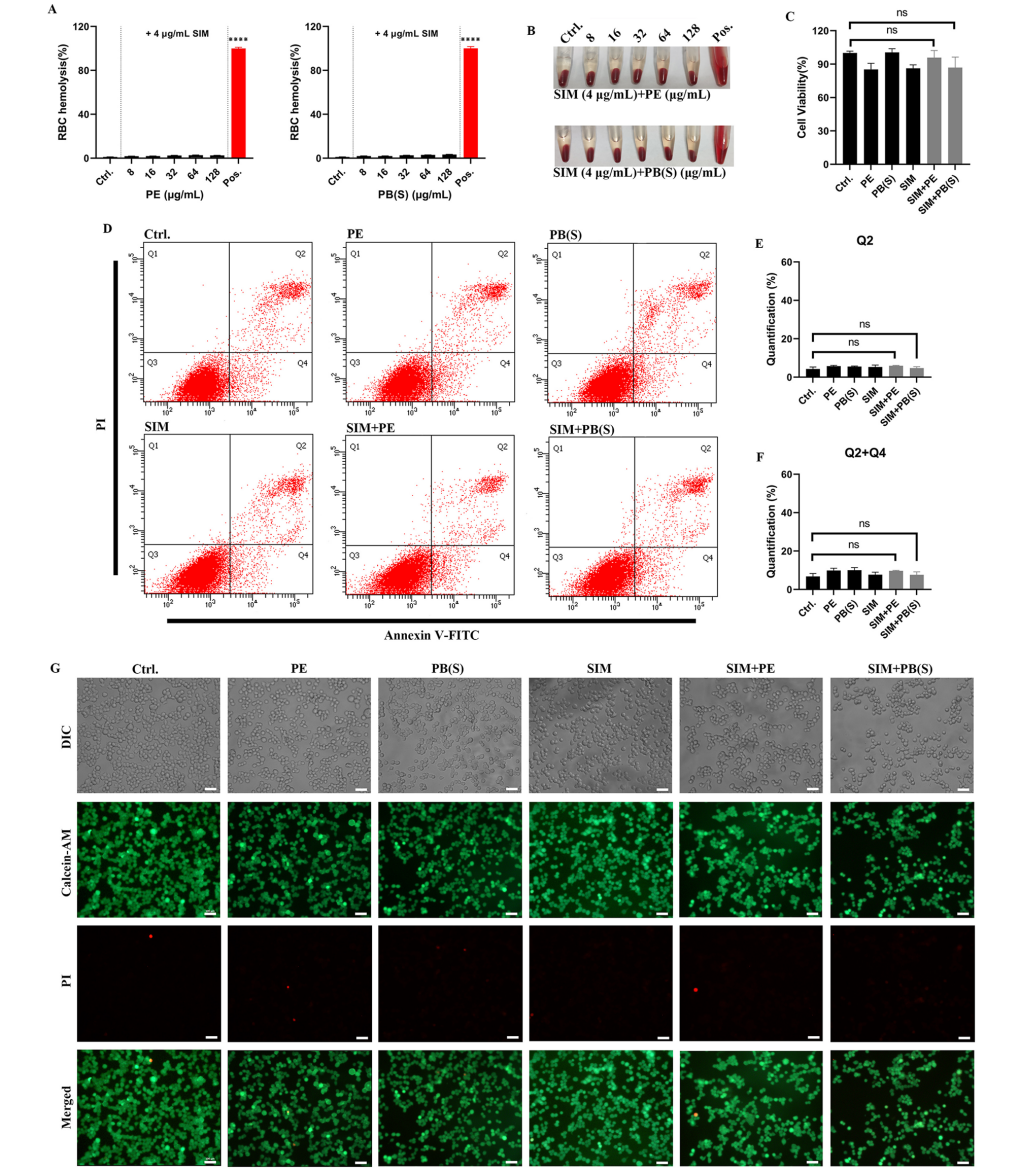
In vitro toxicity assessments of SIM combined with PB(S) or PE. (**A**) Human RBC hemolysis determination after 1 h treatment. (**B**) Representative images of human RBC hemolysis from different groups. (**C**) CCK-8 assay of 293T cells for different drugs treatment [including 32 µg/mL PE, 32 µg/mL PB(S), and 4 µg/mL SIM]; (**D**) Cell apoptosis of 293T cells detected by Annexin V-FITC/PI staining. (**E, F**) Statistical analysis of the apoptotic cell in different groups for Q2 and Q2 + Q4, respectively. (**G**) Representative images of the apoptotic cell observation by Calcein-AM/PI staining. ns: no statistical significance. ****: *P* < 0.0001

In order to investigate the in vitro toxicity of the combination therapy between SIM and polymyxins, we firstly evaluated the human RBC hemolysis effects by PB(S)/PE in the presence of SIM. The results showed that both PB(S) and PE did not cause any hemolysis in human RBC in the presence of 4 µg/mL SIM even at the concentration up to 128 µg/mL (Fig. 3A and B). By CCK-8 assay, the viability of 293T cells treated with 4 µg/mL SIM combined with 32 µg/mL of PB(S)/PE also exhibited no significant difference compared with the untreated group (Fig. 3C). Similarly, SIM and polymyxins combination also exhibited extremely slight toxic to HSF and HaCaT cell lines (Figure S4A). The cell apoptosis induced by SIM combined with PB(S)/PE was detected by Annexin V-FITC/PI staining, as shown in Fig. 3D. As we expected, there was no obvious difference observed between the control and monotherapy groups about the cell counts of Q2 (Fig. 3E), nor in the total number of Q4 + Q2 (Fig. 3F). Further, we visualized the live/dead cells of 293T cells after the treatment by Calcein-AM/PI staining. As shown in Fig. 3G, the viable cells of 293T cells treated with PB(S)/PE and SIM alone or in combination exhibited no obvious change compared with the untreated group. In addition, skin and soft tissue infection (including wound infection and purulent cellulitis, etc.) is one of the most common clinical manifestations of MRSA infection(Hatlen and Miller 2021). Therefore, the toxicity of SIM combined with polymyxins on the effect of skin healing should be assessed by scratch assay. In our study, we found that the migration ability of HaCaT cells in the combination group was not impaired compared with the control or monotherapy groups (Figure S4B; Figure S4C). Collectively, the combination of PB(S)/PE with SIM showed extremely low cytotoxicity to human cell lines.

### SIM combined with polymyxins against high resistant phenotypes

**Fig. 4:**
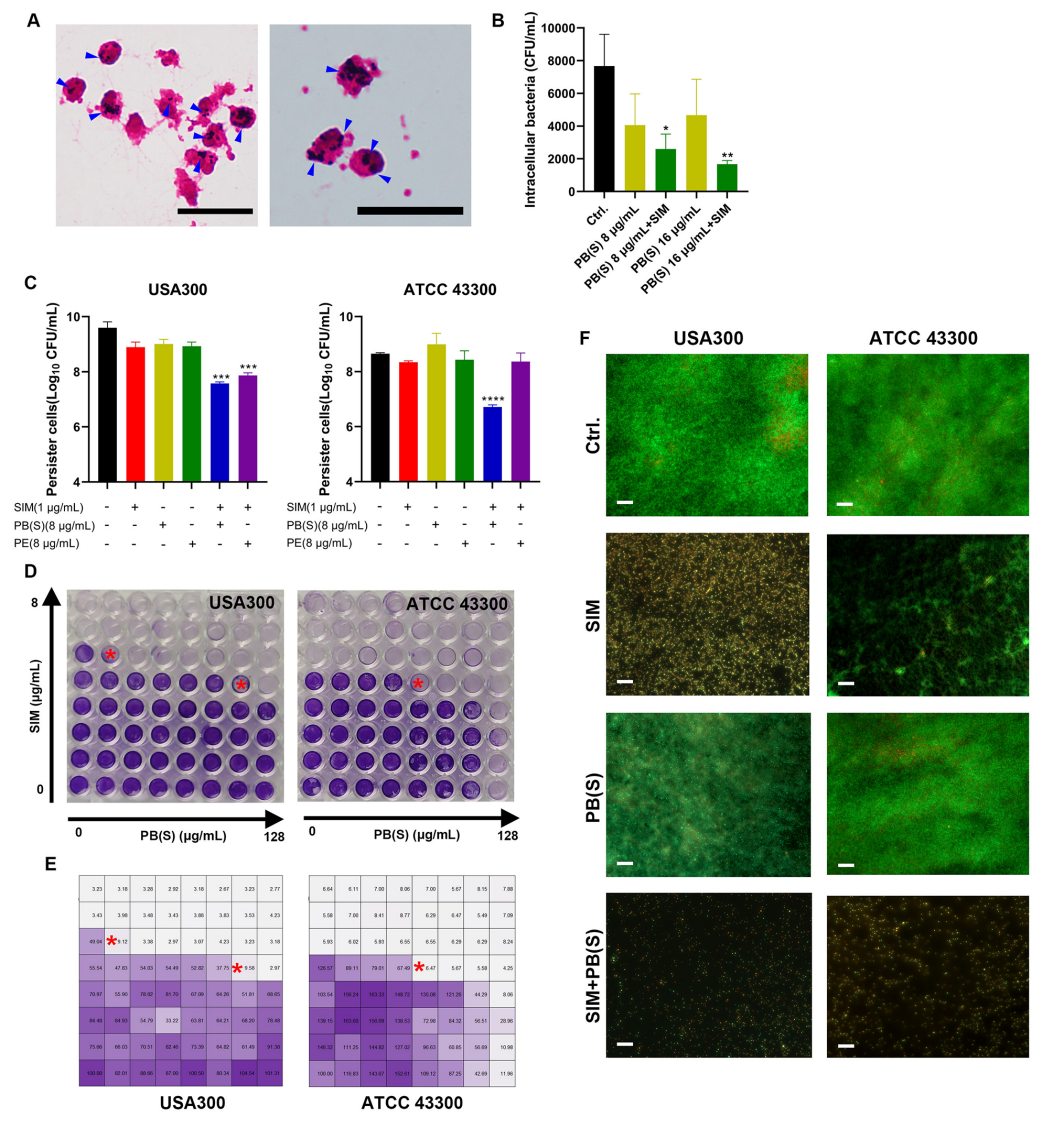
SIM combined with PB(S)/PE against *S. aureus* high resistant phenotypes. (**A**) Phagocytosis of neutrophils against ATCC 43,300. Blue arrows indicate intracellular *S. aureus* cells. Scale: 20 μm. (**B**) Intracellular killing effects of SIM combined with PB(S)/PE against ATCC 43,300. (**C**) Persister cells killing activity by SIM and PB(S)/PE combination. (**D**) Biofilm formation of USA300 and ATCC 43,300 after treated with SIM alone or in combination with PB(S) detected by crystal violet staining. (**E**) Quantification of biofilm formation by crystal violet staining. The asterisks indicate the optimal synergistical biofilm inhibitory concentrations of the tested drugs. (**F**) Biofilm observation by SYTO9/PI staining after treated with SIM alone or in combination with PB(S)/PE [2 µg/mL SIM and 8 µg/mL PB(S) for ATCC 43,300; 1 µg/mL SIM and 16 µg/mL PB(S)] for USA300. Scale: 200 μm. *: *P* < 0.05; **: *P* < 0.01; ***: *P* < 0.001; ****: *P* < 0.0001

Unlike the extracellular bacteria, conventional antibiotics and the host immune system are difficult to remove the intracellular bacteria due to the solid protective barrier provided by neutrophils (Greenlee-Wacker et al. 2014; Li et al. 2021). In our study, we detected the human neutrophils intracellular killing activity of SIM in combination with polymyxins against MRSA. The representative images of intracellular *S. aureus* by Gram staining were shown in Fig. 4A. As we expected, there was a significant reduction in viable CFU counts in the combination group compared with the control or monotherapy group (Fig. 4B). Although, the stationary-phased persister cells showed high resistance to the monotherapy by SIM, PB(S) or PE, 1 µg/mL of SIM significantly enhanced the bactericidal activity of PB(S)/PE against MRSA USA300 persister cells and were decreased to log_10_ 7.57 CFU/mL and log_10_ 7.86 CFU/mL, respectively (Fig. 4C). Similarly, the persister cells counts of ATCC 43,300 between SIM and PB(S) combination was reduced by log_10_ 2.28 CFU/mL compared with the monotherapy group of PB(S). However, no antimicrobial effect was observed against ATCC 43,300 when SIM combined with PE, probably due to the different resistant pattern or bacterial cell components. Further, the crystal violet staining showed that PB(S) alone only showed moderate biofilm inhibition activity against MRSA (ATCC 43,300 and USA300) (Fig. 4D). However, the biofilm inhibition activity of PB(S) was significantly enhanced in the presence of 2 µg/mL SIM. Especially, just 2 µg/mL PB(S) combined with 1 µg/mL SIM achieved significant synergistic inhibition of USA300 biofilm formation (Fig. 4E). As we expected, the thickness and the number of viable bacterial cells in the combinational groups were more obviously reduced than the untreated or monotherapy group by SYTO9/PI staining (Fig. 4F).

### The antimicrobial synergy between SIM and polymyxins was mediated by enhanced membrane disruption

**Fig. 5:**
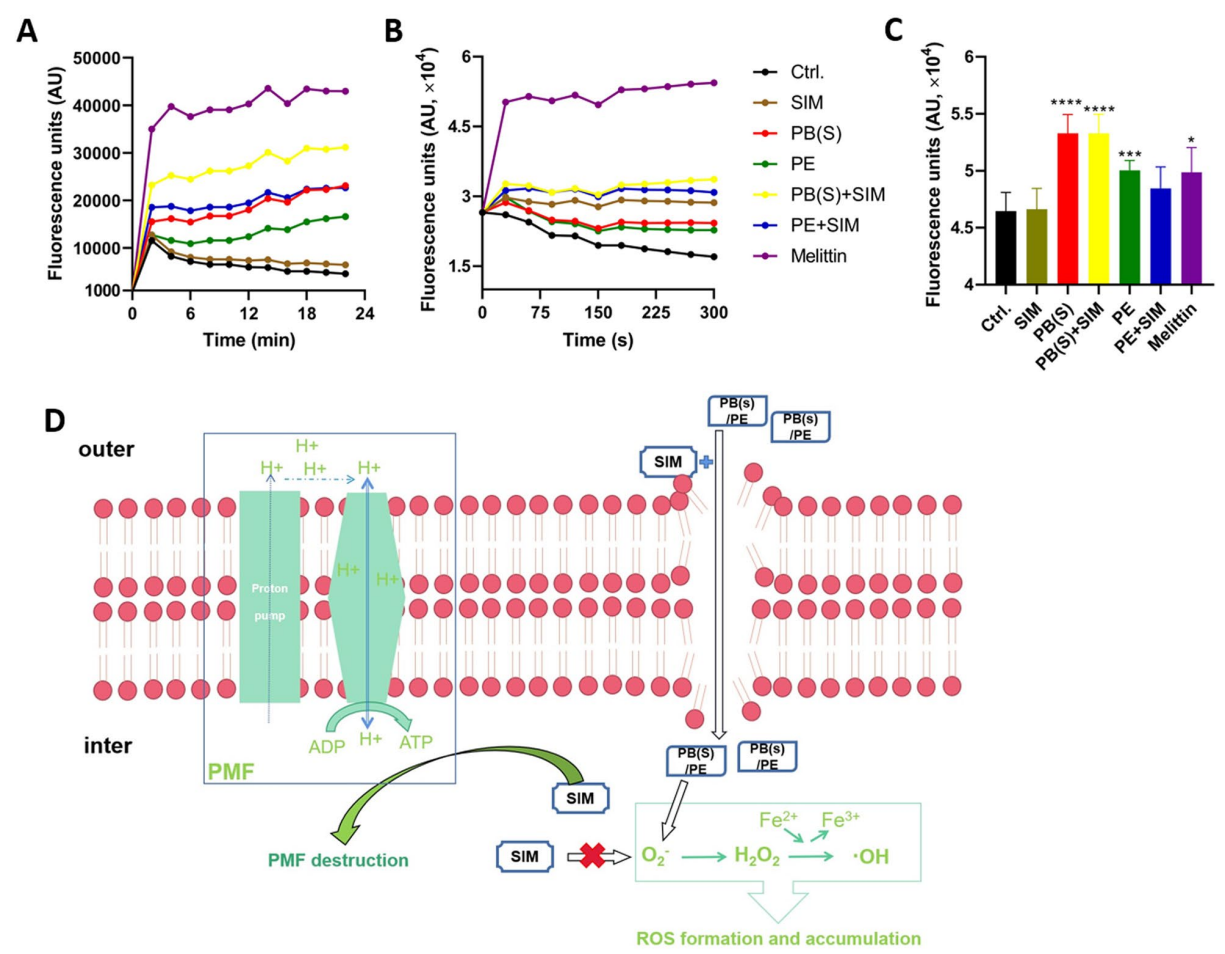
Mechanism of action underline the synergistical combination between SIM and PB(S)/PE. (**A**) Membrane permeabilization determination by SYTOX Green uptake. The *S. aureus* ATCC 43,300 was treated with SIM alone or in combination with PB(S)/PE, 10 µg/mL melittin and DMSO were used as positive and solvent control, respectively. (**B**) Membrane potential determination by DiSC3(5) staining. (**C**) Intracellular ROS detection by DCFH-DA probe. (**D**) Schematic diagram of the possible mechanism by which SIM enhanced the antimicrobial effect of PB(S)/PE. *: *P* < 0.05; ***: *P* < 0.001; ****: *P* < 0.0001

The potential antibacterial mechanism by polymyxins and SIM combination was firstly explored by SYTOX Green probe, which can easily penetrate into the damaged cell membrane, bind to the nucleic acid and further cause an increase number of fluorescence intensity. As shown in Fig. 5A, SIM significantly enhanced the cell plasma membrane disruption activity of PB(S)/PE against ATCC 43,300. Furthermore, we monitored the change of bacterial membrane potential of ATCC 43,300 by using DiSC3(5) probe, and found that the results were consistent with the SYTOX Green assay (Fig. 5B). As reported everywhere, the induction of ROS is one of the main antibacterial pathways of polymyxins (Yin et al. 2020; Yu et al. 2015). Therefore, we found that PB(S)/PE used alone could significantly promote the production of ROS in bacteria, however, the addition of SIM did not increase the accumulation of the ROS by the polymyxins (Fig. 5C). These results suggested that the enhanced antibacterial effect of PB(S)/PE by SIM against MRSA could be related to the increased cell membrane permeability and disruption of membrane potential rather than the enhancement of ROS production (Fig. 5D).

### Synergistic antimicrobial activity between SIM and polymyxins in vivo

**Fig. 6:**
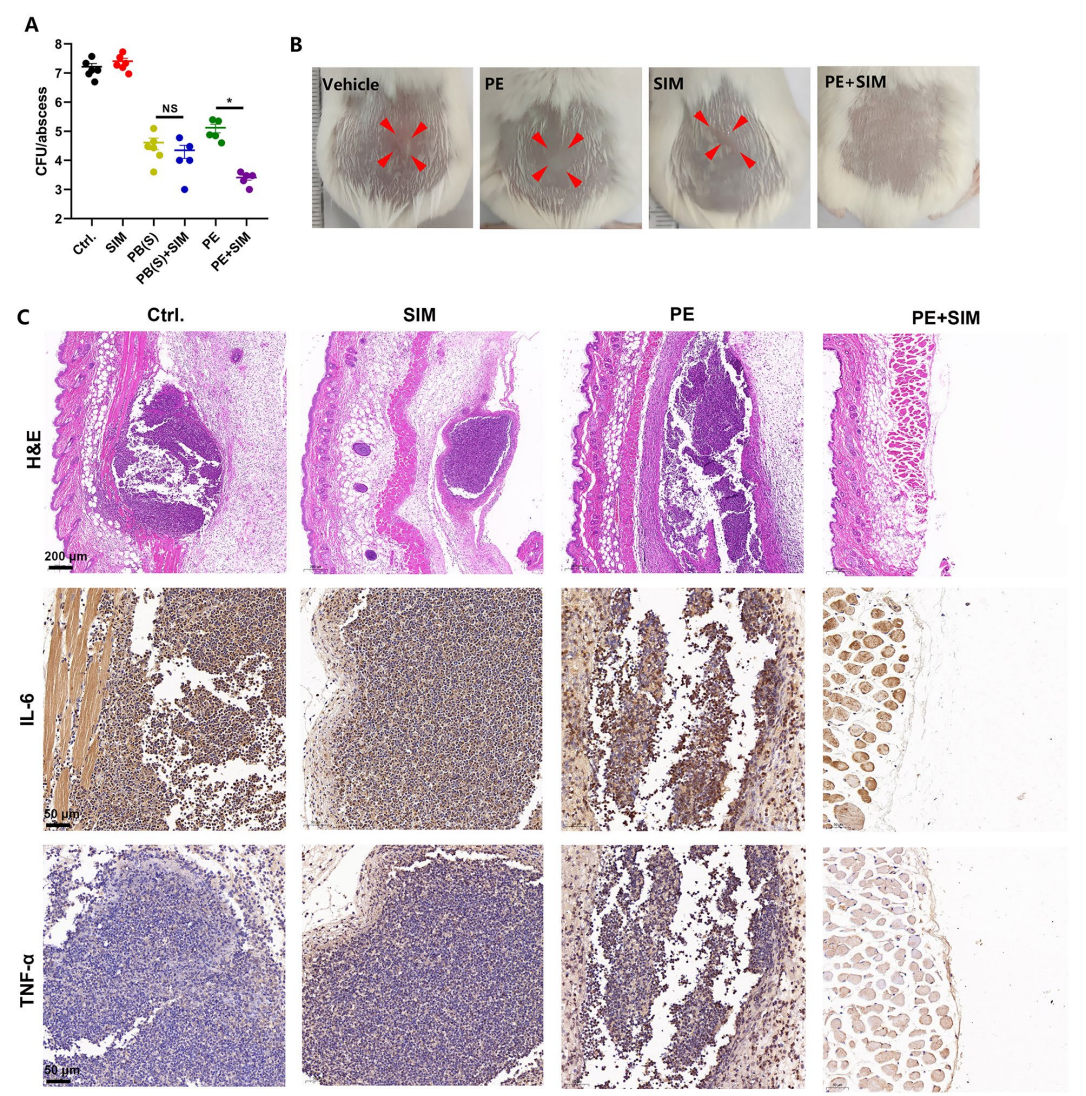
In vivo antimicrobial effect of SIM alone or in combination with PB(S)/PE. (**A**) Viable cell counts in abscess after treatment with PB(S)/PE (30 mg/kg) and SIM (20 mg/kg) alone or in combination. (**B**) Representative images of the abscesses. (**C**) Subcutaneous abscess histopathological analysis using H&E staining and immunohistochemistry of TNF-α or IL-6, respectively. Scale: 200 μm. n = 7 mice per group. ns: no statistical significance. *: *P* < 0.05

We firstly evaluated the toxicity of the drugs in mice. The results of the serum cardiac (CK), hepatic (ALT) and renal (CREA) functional biomarkers showed that there was no significant difference between the combination groups and the vehicle group (Figure S5A). Similarly, H&E staining showed that there were no histopathological changes in myocardial, liver, spleen and kidney between the combination group and control group (Figure S5B). Thus, SIM in combination with PB(S)/PE exhibited extremely low toxicity in vivo. Next, we further explored the antibacterial activity of SIM alone or in combination with PB(S)/PE in vivo. As shown in Fig. 6A, although there was a significant difference between PE used alone and the vehicle group, the SIM combined with PE could synergistically reduce the viable bacterial loads in the abscess (Fig. 6A), which was consistent with the representative images of the subcutaneous abscess in mice (Fig. 6B). However, no significant synergistical antibacterial activity in vivo between the PB(S) and SIM was observed. Because the metabolisms and pharmacokinetics of PE and PB are different (Tran et al. 2016), which could lead to the different in vivo outcomes (Nang et al. 2021; Nation et al. 2014). H&E staining showed the abscess formation and inflammatory cells aggregation in both the vehicle and SIM or PE monotherapy groups, however the abscesses area and the inflammatory cells were largely reduced in the combination treated group (Fig. 6C). Similarly, SIM in combination with PE could also significantly reduced the production of inflammation factors like TNF-α or IL-6 (Fig. 6C).

## Discussion

Abuse of antibiotics has contributed to the worldwide spread of MRSA and made treatments more challenging, which caused an urgent need for new antimicrobial agent development against MRSA (Tong et al. 2015). In our study, we innovatively investigated the antibacterial effect of SIM combined with polymyxins against MRSA, which has not been reported yet. We found that polymyxins, had significant synergistic antibacterial activities against MRSA with SIM in vitro, among which PE combined with SIM had a significant antibacterial effect in vivo. In addition, we preliminarily found that SIM strongly enhanced the bacterial cell membrane disruption by PB(S)/PE. The synergistical effectiveness of SIM in combination with PB(S)/PE against high resistant phenotypes of *S. aureus* suggested its potential as an optional treatment for chronic infection caused by biofilm or persister cells.

The absence of LPS and the presence of a physical barrier formed by the thick peptidoglycan layer prevent polymyxins from penetrating the cell membrane of Gram-positive bacteria (Yin et al. 2020). However, polymyxins display the potential to disrupt the cell membrane leading to cell death when combined with adjuvants. The combination of PBT2, Zinc and colistin showed bactericidal activity against Gram-positive bacteria (Oliveira et al. 2022), which breaks its intrinsic polymyxin resistance. Similarly, we also found that the presence of sub-MIC of SIM restored the antibacterial activity of PB(S)/PE against *S. aureus* through the antimicrobial susceptibility test, checkerboard assay and time-growth inhibition curve, etc. Furthermore, various studies have demonstrated that polymyxins have enhanced antimicrobial activities against Gram-negative pathogens when combined with antibiotic adjuvants such as econazole (Xie et al. 2022), nisin (Thomas et al. 2019) and otilonium (Xu et al. 2022), etc. Therefore, in our study, polymyxins combined with SIM as an adjuvant will become a broad-spectrum antibiotic for the treatment of *S. aureus* infection to increase clinical application.

It is reported that there were only reversible bilirubin elevations in organic function biomarkers and mild adverse events (including fatigue, nausea, fatigue and diarrhea et al.) in HCV genotype-1 patients received 200 mg dose of SIM (Manns et al. 2011). In addition, extremely low incidence of clinical adverse events (including rash and anemia) was reported when SIM was in combination with PEGylated interferon and ribavirin (You and Pockros 2013), which indicated the good safety and tolerability of SIM. In addition, nephrotoxicity and neurotoxicity were the most prominent toxicities associated with polymyxins (Nang et al. 2021), and reducing the daily dose by addition of SIM may reduce the risk of toxicity. In our study, the combination was found to have less in vitro cytotoxicity, and 30 mg/kg/day PB(S)/PE in combination with 20 mg/kg/day SIM were also exhibited undetectable toxicity in mice in vivo. These results suggest that SIM combined polymyxins may be a potential therapy for MRSA infection with acceptable toxicity profile.

The formation of biofilm (Nasser et al. 2022)as well as the appearance of persister cells (Fisher et al. 2017) are common causes of high resistance of *S. aureus* to antibiotics, further leading to the presence of chronic and recurrent infection, thus increasing the difficulty of treatment for bacterial infection in clinic. We found that SIM combined with PB(S)/PE can effectively inhibit the formation of biofilm, and have an effective bactericidal activity against persister cells, which suggests that the combination is beneficial to control the recurrent infection caused by MRSA.

Previous studies have reported that polymyxins achieved antibacterial effects mainly through: (1) binding to negatively charged LPS to destroy the outer membrane and lysing the inner membrane, further resulting in bacterial death (Mohapatra et al. 2021; Sabnis et al. 2021); (2) leading to cell lysis by promoting phospholipid exchange between internal phospholipid vesicles of outer membrane and inner membrane (Cajal et al. 1996); (3) inducing continuous accumulation of ROS through Fenton reaction, further inducing rapid cell death by causing oxidative damage of DNA, lipid and protein (Ayoub Moubareck 2020; Kohanski et al. 2007; Yu et al. 2015). In addition, Rudilla et al. (Rudilla et al. 2018) performed isothermal titration calorimetry experiments to find that polymyxin-like cationic peptides can react with teichoic acid in a three-step to kill Gram-positive bacteria and promote cell death through oxidative damage. Our previous study has preliminarily explored the antibacterial mechanisms of SIM against *S. aureus* by the disruption of cell membrane permeability. And we also found SIM could interfere with the synthesis of ATP through the destruction of proton-motive force (Li et al. 2022). Similarly, in this study, we also found that the presence of sub-MIC SIM could synergistically enhanced the destruction of bacterial cell membrane by PB(S)/PE. And this could be the underlying mechanism of the synergy between SIM and PB(S)/PE against *S. aureus* (Fig. 5D). However, no synergistic antibacterial activity against Gram-negative strains was observed by SIM in combination with PB(S)/PE. This could be due to the negatively charged SIM repelled by the negatively charged LPS, so that SIM could not penetrate into the Gram-negative bacteria.

In summary, SIM restored the anti-*Staphylococcus* activity of polymyxins in vitro and in vivo. SIM combined with PB(S)/PE also exhibited effective bactericidal activities against high resistant phenotypes of intracellular bacteria, persister cells and biofilms. Low cytotoxicity and in vivo toxicity indicated the applicability of this combination. These results suggest that SIM is a promising adjuvant to repurposing polymyxins as broad-spectrum antibiotics.

### Electronic supplementary material

Below is the link to the electronic supplementary material.

Supplementary Material 1

## Data Availability

Date will be made available on request.
