# Supplementary material for: Simeprevir restores the anti-Staphylococcus activity of polymyxins
Source: AMB Express. 2023 Nov 2;13:122. doi: 10.1186/s13568-023-01634-8 (PMC10622387; doi:10.1186/s13568-023-01634-8)
Supplement: Supplementary file 1 — Supplementary Material 1 [file 13568_2023_1634_MOESM1_ESM.docx]

## Supplemental Material

## Supplementary Figures


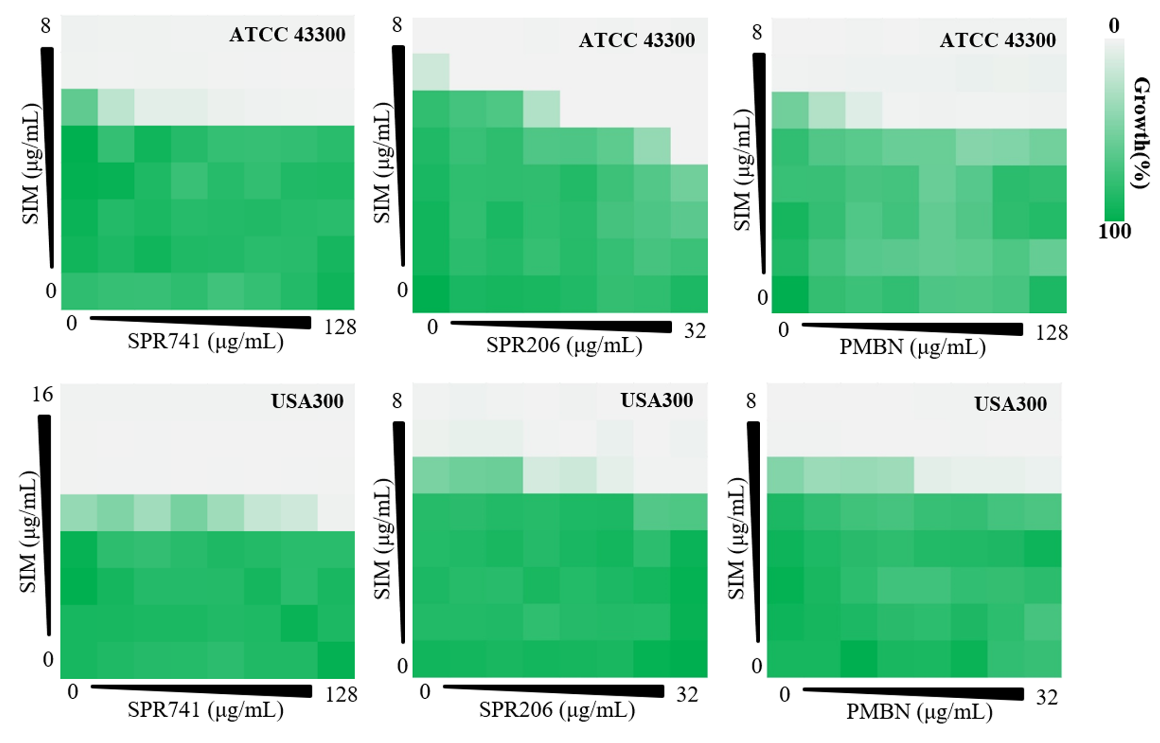


**Figure S1.** Drug combination between SIM and polymyxins against *S. aureus* by checkerboard assay.


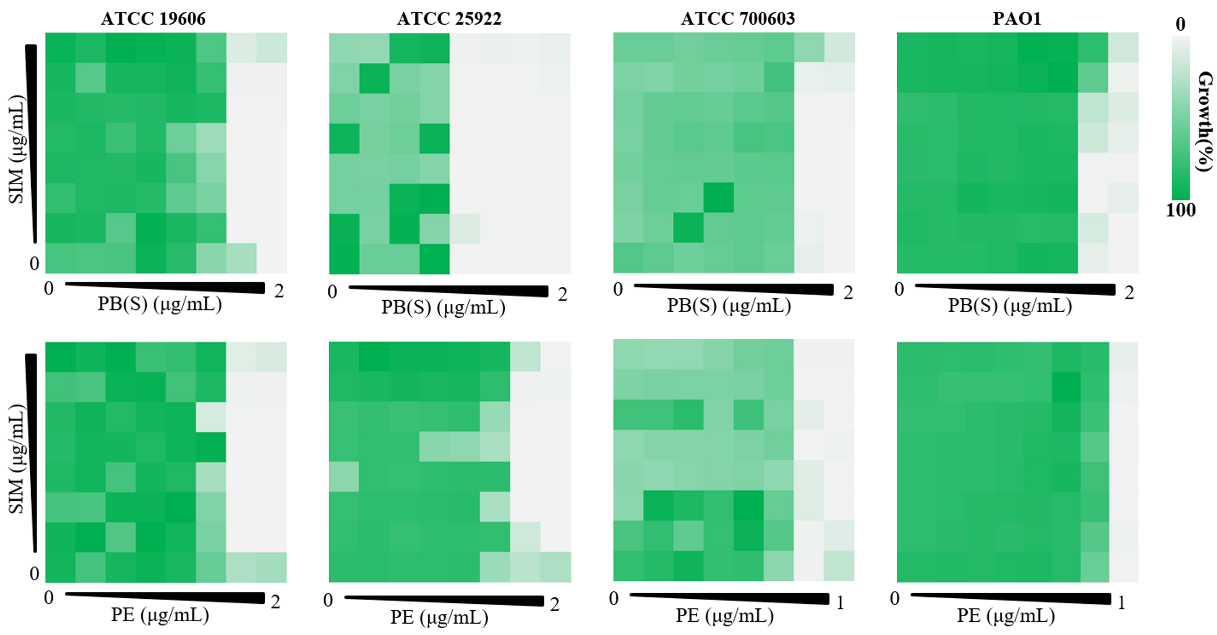


**Figure S2.** Drug combination between SIM and PB(S)/PE against Gram-negative bacteria by checkerboard assay (MICs of PE against ATCC 700603/PAO1 are 1 μg/mL, MICs of PB(S) against ATCC 700603/PAO1 are 2 μg/mL, and PE/PB(S) against ATCC 25922/ATCC 19606 are 2 μg/mL.)


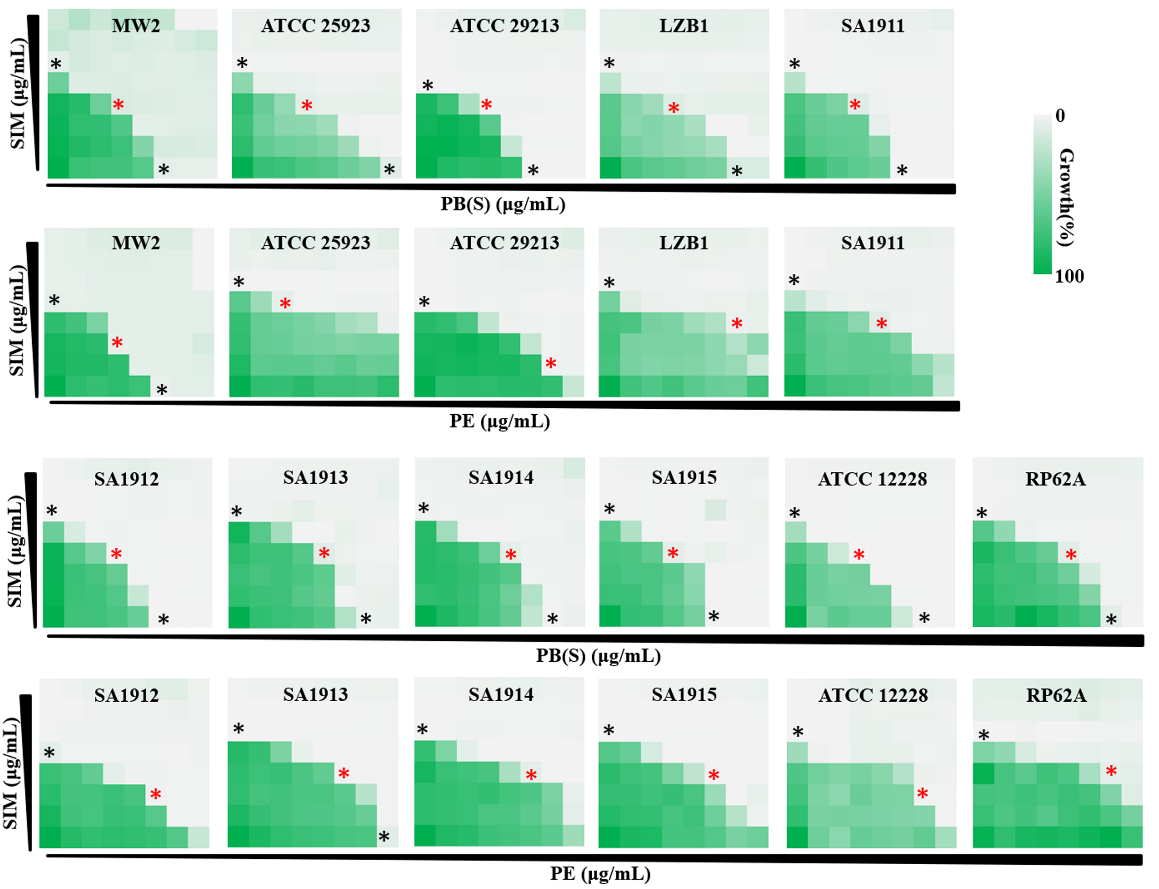


**Figure S3**. Drug combination between SIM and PB(S)/PE against *Staphylococcus* type strains or clinical isolates (MRSA, MSSA, and *S. epidermidis*). The concentration of SIM is 0-8 μg/mL, the concentration of PB(S) and PE were both 0-128 μg/mL. If MIC>128 μg/mL, assume MIC=256 μg/mL to obtain the assumed maximum FICI value. The asterisks indicate the calculation of FICI.


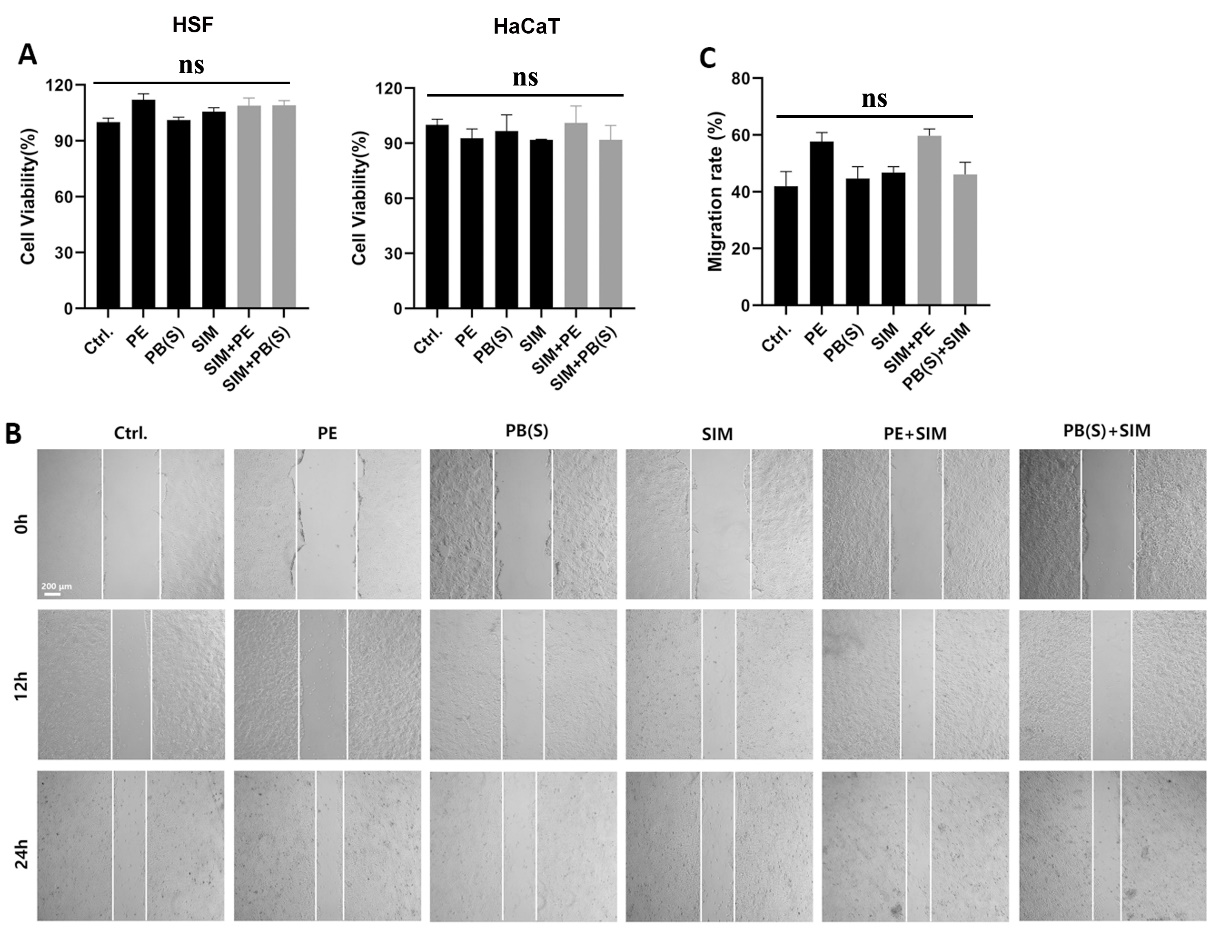


**Figure S4**. Cytotoxicity of SIM and PE/PB(S) combinations. (A) Cytotoxicity of HSF and HaCaT determined by CCK-8, respectively. (B) Scratch assay of HaCaT for 24h treatment. SIM: 2 μg/mL; PB(S): 32 μg/mL; PE: 32 μg/mL. Scale: 200 μm. (C) Migration rate (%) of HaCaT. ns: no statistical significance.


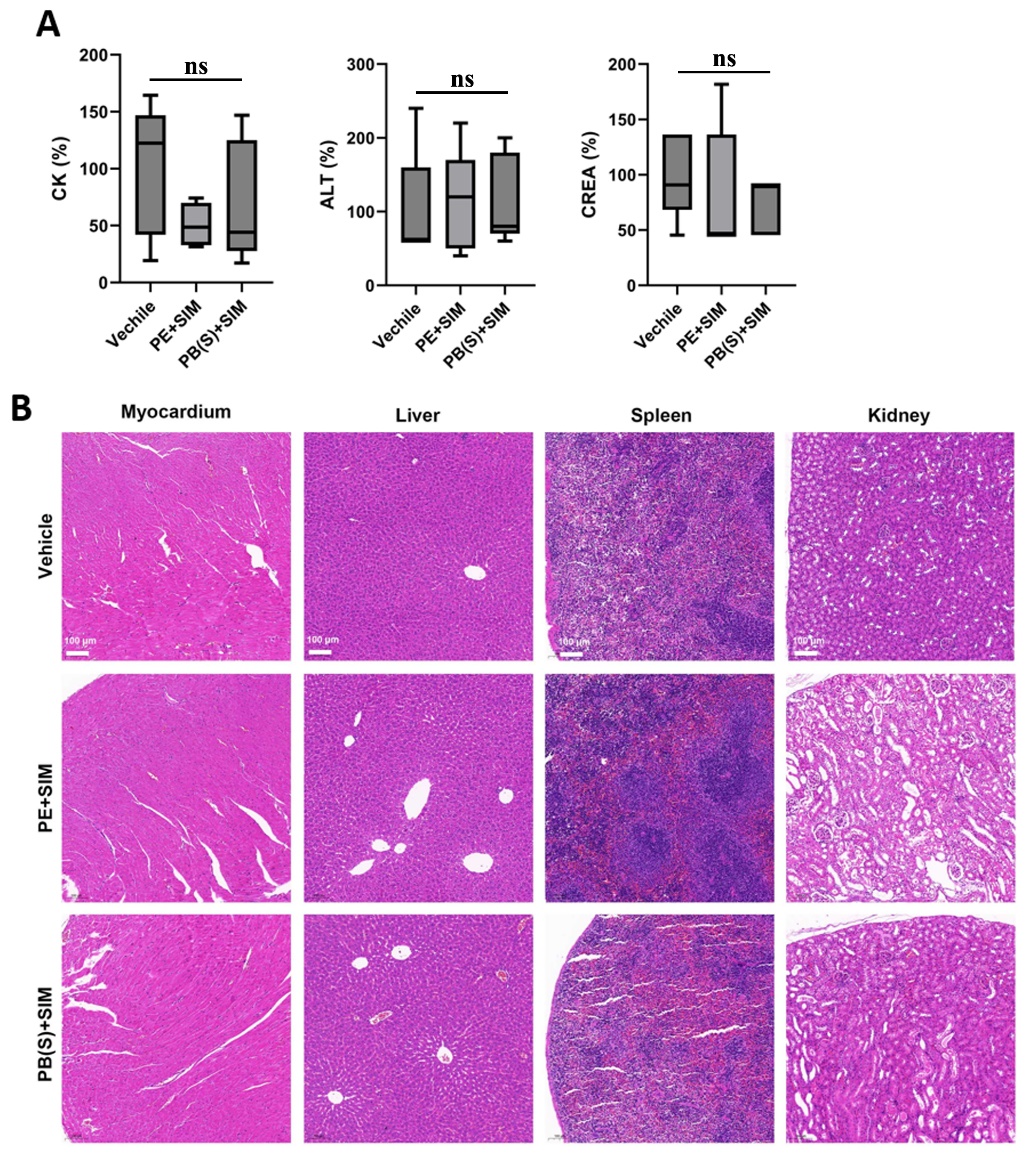


**Figure S5**. *In vivo* toxicity of SIM and PE/PB(S) combination. (A) Serum CK, ALT and CREA quantification. (B) H&E staining (from left to right: myocardium, liver, spleen and kidney.) Scale: 100 µm. n = 7 mice per group. ns: no statistical significance.
